# Corneal Confocal Microscopy detects a Reduction in Corneal Endothelial Cells and Nerve Fibres in Patients with Acute Ischemic Stroke

**DOI:** 10.1038/s41598-018-35298-3

**Published:** 2018-11-26

**Authors:** Adnan Khan, Saadat Kamran, Naveed Akhtar, Georgios Ponirakis, Hamad Al-Muhannadi, Ioannis N. Petropoulos, Shumoos Al-Fahdawi, Rami Qahwaji, Faheem Sartaj, Blessy Babu, Muhammad Faisal Wadiwala, Ashfaq Shuaib, Rayaz A. Malik

**Affiliations:** 1 Department of Medicine, Weill Cornell Medicine-Qatar, Doha, Qatar; 2 Department of Neurology and Institute of Neurosciences, Hamad Medical Corporation, Doha, Qatar; 3 School of Electrical Engineering and Computer Science, University of Bradford, Bradford, UK; 4 Stroke Program, Department of Neurology, University of Alberta, Edmonton, Canada

## Abstract

Endothelial dysfunction and damage underlie cerebrovascular disease and ischemic stroke. We undertook corneal confocal microscopy (CCM) to quantify corneal endothelial cell and nerve morphology in 146 patients with an acute ischemic stroke and 18 age-matched healthy control participants. Corneal endothelial cell density was lower (*P* < 0.001) and endothelial cell area (*P* < 0.001) and perimeter (*P* < 0.001) were higher, whilst corneal nerve fibre density (*P* < 0.001), corneal nerve branch density (*P* < 0.001) and corneal nerve fibre length (*P* = 0.001) were lower in patients with acute ischemic stroke compared to controls. Corneal endothelial cell density, cell area and cell perimeter correlated with corneal nerve fiber density (*P* = 0.033, *P* = 0.014, *P* = 0.011) and length (*P* = 0.017, *P* = 0.013, *P* = 0.008), respectively. Multiple linear regression analysis showed a significant independent association between corneal endothelial cell density, area and perimeter with acute ischemic stroke and triglycerides. CCM is a rapid non-invasive ophthalmic imaging technique, which could be used to identify patients at risk of acute ischemic stroke.

## Introduction

The major risk factors for stroke include diabetes, hypertension, smoking, dyslipidemia^1–5^ and metabolic syndrome^6^. Endothelial dysfunction is a key underlying abnormality in stroke and in those at risk of stroke, by promoting vasoconstriction and enhanced plaque vulnerability and rupture, with thrombus formation^7^. Endothelial dysfunction can be assessed using a variety of techniques including brachial flow-mediated dilation, cerebrovascular reactivity to L-arginine and laser Doppler^8^. Indeed we have previously shown impaired endothelium dependent dilatation in patients with obesity^9^, diabetes and hypertension^10^ and an association between small artery remodeling and diastolic dysfunction in obese subjects^11^. Patients admitted with an acute ischemic stroke have reduced forearm flow mediated dilatation and increased circulating levels of P-selectin, a marker of endothelial dysfunction^12^. Direct imaging of the cerebral blood vessels can identify atherosclerosis and stenosis^13^ and brain imaging can identify silent infarcts, cerebral microbleeds, periventricular white matter hyperintensities and perivascular spaces, which all predict a higher risk of stroke^14,15^. Subtle alterations in the microstructure of normal-appearing white matter also predicts stroke^16^. Retinal vessel dysfunction and altered structure have been related to cardiovascular disease^8,17^, stroke^18^ and recurrent stroke^19^.

The major function of the corneal endothelium is to regulate corneal hydration and the passage of nutrients and metabolic waste to and from stromal keratocytes^20^. However, it produces comparable type and amount of extracellular matrix and collagen to aortic and venous endothelium^21^, and exposure of corneal endothelial cells to fibrin^22^ or thrombin^23^ leads to the induction of tissue-plasminogen activator. Non-contact specular microscopy has been used to identify a reduction in corneal endothelial cell density and increased polymegathism in some studies of patients with Type 2 diabetes^24^ and children with Type 1 diabetes^25^, but not in others^26^.

Corneal confocal microscopy is a rapid non-invasive ophthalmic imaging technique that demonstrates corneal nerve damage in patients with diabetic and HIV neuropathy^27,28^, Parkinson’s disease^29^, multiple sclerosis^30,31^ and acute ischemic stroke^32^. We have also previously demonstrated a reduction in corneal endothelial cell density in patients with Type 1 diabetes^33^ and Type 2 diabetes^34^.

In the present study, we have utilized CCM to quantify corneal endothelial cell and nerve morphology in patients with acute ischemic stroke.

## Results

### Clinical and Metabolic parameters

The clinical and laboratory characteristics of the participants are given in Table 1. One hundred and forty-six patients with acute ischemic stroke, with (HbA_1c _ ≥  6.5%) (n = 50) and without (HbA_1c_ ≤ 6.4%) (n = 96) type 2 diabetes mellitus (T2DM) were compared with 18 age-matched healthy control participants. The duration of diabetes in diabetic patients with ischemic stroke was 7.94 ± 7.50 years. There were no differences in age, BMI, total cholesterol, LDL and HDL between controls and stroke patients. Stroke patients had higher triglycerides (*P* = 0.05), HbA_1c_ (*P* < 0.04), systolic blood pressure (*P* < 0.001) and diastolic blood pressure (*P* < 0.001) compared to control participants (Table 1).

**Table 1:** Clinical metabolic and corneal endothelial and nerve parameters in control subjects and patients with acute ischemic stroke.

Variables	Controls	Stroke	*P* value
Number of Participants	18	146	
Age (years)	47.73 ± 3.10	48.93 ± 0.79	0.714
Gender (M/F)	(11/7)	(141/5)	<0.001
BMI (kg/m^2^)	25.78 ± 0.63	29.40 ± 0.83	0.217
NIHSS Score	N/A	4.08 ± 0.33	NA
Triglycerides (mmol/l)	1.23 ± 0.24	1.86 ± 0.10	0.053
Total Cholesterol (mmol/l)	4.63 ± 0.35	5.05 ± 0.10	0.337
LDL (mmol/l)	2.96 ± 0.33	3.27 ± 0.09	0.421
HDL (mmol/l)	1.10 ± 0.07	0.94 ± 0.02	0.058
BP Systolic (mmHg)	120.40 ± 3.96	161.03 ± 2.47	<0.001
BP Diastolic (mmHg)	73.60 ± 2.44	94.10 ± 1.41	<0.001
HbA_1c_ (%)	5.36 ± 0.17	6.83 ± 0.18	0.035
Diabetes Duration (years)	NA	7.94 ± 7.50	NA
Mean ECD (no./mm^2^)	3664.72 ± 43.88	3342.87 ± 27.45	<0.001
Mean ECA (µm^2^)	219.81 ± 2.69	244.37 ± 2.05	<0.001
Mean ECP (µm)	52.95 ± 0.35	55.74 ± 0.25	<0.001
Polymegathism (%)	52.26 ± 1.31	52.39 ± 0.44	0.923
Pleomorphism (%)	33.51 ± 1.21	33.60 ± 0.50	0.953
CNFD (no./mm^2^)	37.54 ± 1.97	28.73 ± 0.65	<0.001
CNBD (no./mm^2^)	73.96 ± 6.15	49.35 ± 2.26	< 0.001
CNFL (mm/mm^2^)	21.31 ± 1.01	16.92 ± 0.42	0.001

BMI (Body Mass Index), NIH stroke severity (NIHSS), LDL (Low Density Lipoprotein), HDL (High Density Lipoprotein), BP (Blood Pressure), HbA_1c_ (Glycated hemoglobin), mean ECD (Endothelial Cell Density), mean ECA (Endothelial Cell Area), mean ECP (Endothelial Cell Perimeter), CNFD (Corneal nerve fibre density), CNBD (Corneal nerve branch density), CNFL (Corneal nerve fibre length). Results are expressed as mean ± SE with significance indicated by the exact *P* value.

### Corneal Confocal Microscopy

#### Corneal Endothelium

**Figure 1:**
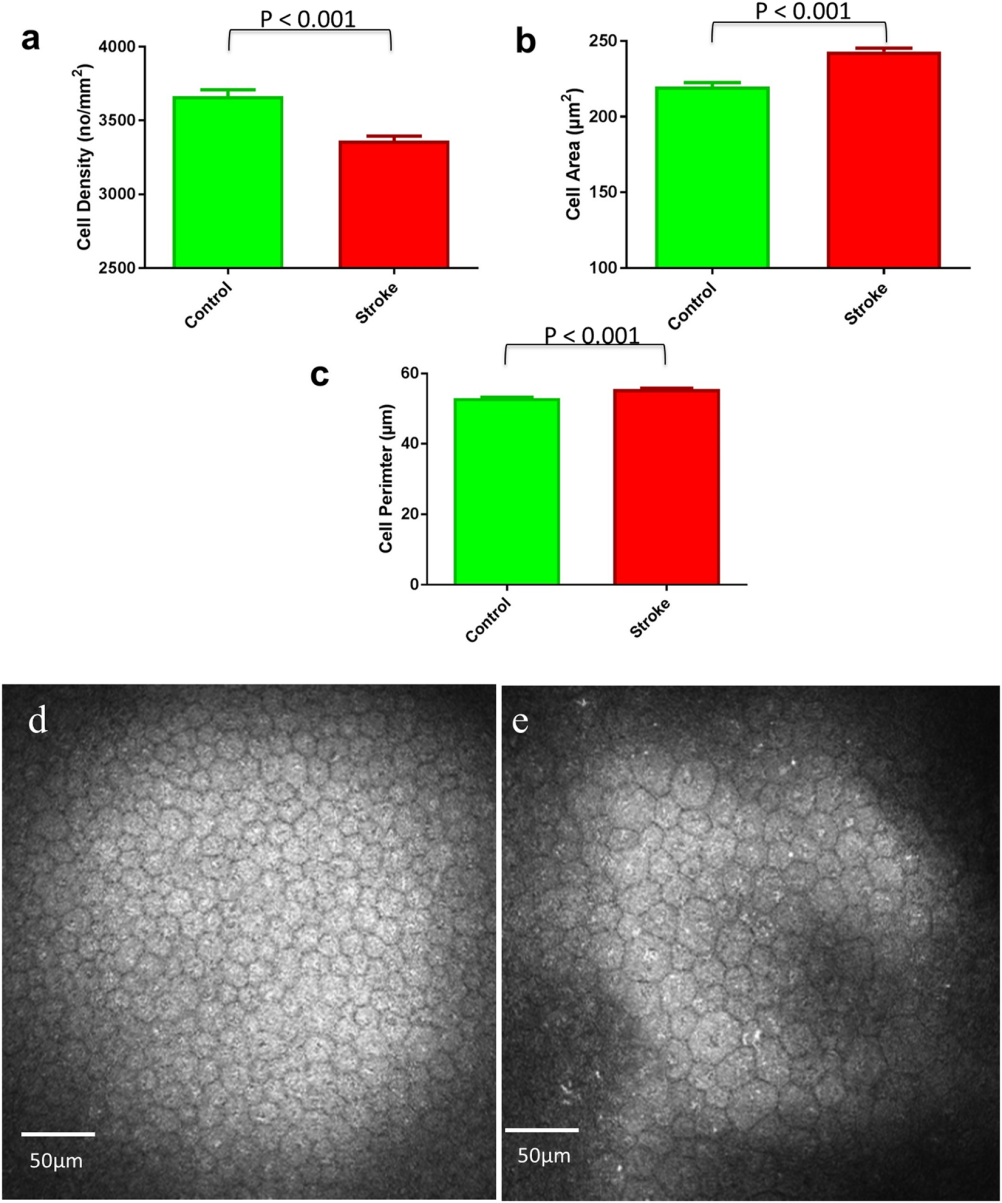
Graphs showing endothelial cell density (**a**), endothelial cell area (**b**) and endothelial cell perimeter (**c**) expressed as Mean and SEM in participants with acute ischemic stroke and control subjects and an image of corneal endothelial cells in a control participant (**d**) and a patient with acute ischemic stroke (**e**).

Corneal endothelial cell density was lower (*P* < 0.001) and endothelial cell area (*P* < 0.001) and perimeter (*P* < 0.001) were higher, but there were no significant difference in the percentage polymegathism and pleomorphism in stroke patients compared to healthy controls (Table 1; Fig. 1).

There was no significant difference in corneal endothelial cell density (3363.87 ± 34.45; 3302.55 ± 45.16, *P* = 0.283), area (242.90 ± 2.56; 247.19 ± 3.40, *P* = 0.322), perimeter (55.53 ± 0.31; 56.14 ± 0.41, *P* = 0.247), polymegathism (52.35 ± 0.57; 52.45 ± 0.70, *P* = 0.920) or pleomorphism (33.61 ± 0.61; 33.59 ± 0.87, *P* = 0.985) in patients with and without diabetes, respectively.

#### Corneal Nerves

Corneal nerve fibre density (*P* < 0.001), corneal nerve branch density (*P* < 0.001) and corneal nerve fibre length (*P* = 0.001) were lower in patients with acute ischemic stroke compared to controls (Table 1).

#### Correlation between endothelial cell and nerve morphology

In all stroke patients, corneal endothelial cell density correlated with corneal nerve fiber density (r = 0.177, *P* = 0.033) and corneal nerve fiber length (r = 0.199, *P* = 0.017). Endothelial cell area and perimeter correlated with corneal nerve fiber density (r = −0.204, *P* = 0.014, r = −0.211, *P* = 0.011) and corneal nerve fiber length (r = −0.207, *P* = 0.013, r = −0.220, *P* = 0.008), respectively (Table 2). There was no significant correlation between corneal endothelial cell parameters and corneal nerve branch density or between % polymegathism and pleomorphism and corneal nerve parameters.

**Table 2:** Correlation between endothelial cell and corneal nerve parameters in patients with ischemic stroke, with significant values in bold.

Variables	CNFD	CNFL	CNBD
Endothelial Cell Density			
Coefficient (r)	**0.177**	**0.199**	0.116
*P*	**(0.033)**	**(0.017)**	(0.166)
Endothelial Cell Area			
Coefficient (r)	−**0.204**	−**0.207**	−0.128
*P*	**(0.014)**	**(0.013)**	(0.125)
Endothelial Cell Perimeter			
Coefficient (r)	−**0.211**	−**0.220**	−0.140
*P*	**(0.011)**	**(0.008)**	(0.093)
Polymegathism			
Coefficient (r)	−0.082	−0.018	−0.054
*P*	(0.327)	(0.831)	(0.515)
Pleomorphism			
Coefficient (r)	0.093	0.068	0.092
*P*	(0.263)	(0.416)	(0.271)

ECD (Endothelial Cell Density), ECA (Endothelial Cell Area), ECP (Endothelial Cell Perimeter), CNFD (Corneal nerve fibre density), CNBD (Corneal nerve branch density), CNFL (Corneal nerve fibre length).

In stroke patients without diabetes, corneal endothelial cell density correlated with corneal nerve fiber density (r = 0.208, *P* = 0.042). Endothelial cell area and perimeter correlated inversely with corneal nerve fiber density (r = −0.241, *P* = 0.018, r = −0.236, *P* = 0.021) and corneal nerve fiber length (r = −0.207, *P* = 0.037, r = −0.216, *P* = 0.0035), respectively (Supplementary Table 1). There was no significant correlation between corneal endothelial cell parameters and CNBD or between % polymegathism and pleomorphism and corneal nerve parameters. In stroke patients with diabetes, there was no significant correlation between endothelial cell density, cell area or perimeter and corneal nerve parameters. Endothelial cell pleomorphism correlated with CNFD (r = 0.309, *P* = 0.031) and polymegathism correlated with corneal nerve fiber density (r = −0.373, *P* = 0.008), corneal nerve fiber length (r = −0.296, *P* = 0.039) and corneal nerve branch density (r = −0.334, *P* = 0.019) (Supplementary Table 2).

#### Multiple Linear Regression

There was an independent association between endothelial cell density and triglycerides (P = 0.05) (Table 3). Endothelial cell area was independently associated with higher triglycerides (P = 0.04) and acute ischemic stroke (P = 0.05) (Table 4). Endothelial cell perimeter was independently associated with higher triglycerides (P = 0.04) and acute ischemic stroke (P = 0.05) (Table 5).

**Table 3:** Estimates of endothelial cell density and independent variables in multiple regression with significance.

Parameter	Estimate	95% CI Lower Bound	95% CI Upper Bound	Standard Error	Significance level P Value
**Dependent Variable: Endothelial Cell Density**					
Constant	3707.505	3127.029	4287.980	293.492	<0.001
Age	−3.873	−10.062	2.315	3.129	0.218
BMI	−3.159	−8.863	2.545	2.884	0.275
Triglycerides	−95.066	−191.861	1.729	48.940	**0.054**
Cholesterol	144.913	−67.325	357.152	107.309	0.179
LDL	−110.805	−329.658	108.049	110.654	0.318
HDL	−269.492	−572.551	33.567	153.228	0.081
Systolic BP	−1.174	−3.895	1.547	1.376	0.395
Diastolic BP	4.362	−0.418	9.143	2.417	0.073
HbA_1c_	5.169	−20.947	31.285	13.204	0.696
Stroke	−277.299	−595.120	40.523	160.692	**0.087**

**Table 4:** Estimates of endothelial cell area and independent variables in multiple regression with significance.

Parameter	Estimate	95% CI Lower Bound	95% CI Upper Bound	Standard Error	Significance level P Value
Dependent Variable: Endothelial Cell Area					
Constant	217.302	174.154	260.45	21.816	<0.001
Age	0.323	−0.137	0.783	0.233	0.167
BMI	0.202	−0.222	0.626	0.214	0.348
Triglycerides	7.564	0.369	14.759	3.638	**0.039**
Cholesterol	−12.025	−27.801	3.752	7.977	0.134
LDL	9.956	−6.312	26.224	8.225	0.228
HDL	19.112	−3.415	41.639	11.39	0.096
Systolic BP	0.086	−0.117	0.288	0.102	0.403
Diastolic BP	−0.337	−0.692	0.018	0.18	0.063
HbA_1c_	−0.679	−2.621	1.262	0.982	0.49
Stroke	23.883	0.258	47.507	11.945	**0.048**

**Table 5:** Estimates of endothelial cell perimeter and independent variables in multiple regression with significance.

Parameter	Estimate	95% CI Lower Bound	95% CI Upper Bound	Standard Error	Significance level P Value
**Dependent Variable: Endothelial Cell Perimeter**					
Constant	52.7	47.456	57.943	2.651	0.001
Age	0.035	−0.021	0.091	0.028	0.218
BMI	0.025	−0.026	0.077	0.026	0.330
Triglycerides	0.893	0.018	1.767	0.442	**0.045**
Cholesterol	−1.313	−3.23	0.604	0.969	0.178
LDL	1	−0.977	2.977	0.999	0.319
HDL	2.271	−0.467	5.008	1.384	0.103
Systolic BP	0.009	−0.016	0.033	0.012	0.487
Diastolic BP	−0.041	−0.084	0.002	0.022	0.063
HbA_1c_	−0.052	−0.288	0.184	0.119	0.666
Stroke	2.933	0.062	5.803	1.451	**0.045**

## Discussion

This is the first study to show a reduction in corneal endothelial cell density and an increase in endothelial cell size in patients with acute ischemic stroke. A study in Type 2 diabetic rats has shown impaired posterior ciliary artery relaxation and corneal nerve loss, suggesting that impaired blood flow to the trigeminal ganglion may be related to corneal nerve loss^35^. In the present study, we show a modest but significant correlation between the change in corneal endothelial cells and loss of corneal nerves. However, a correlation cannot imply cause and effect and common underlying abnormalities could drive both corneal endothelial cell and nerve fibre abnormalities. Indeed Olsen previously showed a higher prevalence of ischemic heart disease in patients with Fuch’s dystrophy and suggested that endothelial dystrophy and atherosclerosis may have common mechanisms^36^. Additionally, a number of studies of patients with corneal endothelial dystrophies have demonstrated a reduction in corneal nerve fibres^37^. Conversely, patients with neurotrophic keratitis and hence a primary loss of corneal nerve fibres have been shown to have endothelial cell abnormalities^37,38^. Furthermore, corneal nerve loss has been related to a progressive reduction in corneal endothelial cells in patients with dry eye disease^39^.

Diabetes, hypertension, smoking, dyslipidemia^1–5,40,41^, obesity and metabolic syndrome^6,42^ lead to endothelial dysfunction and atherosclerosis and are major risk factors for stroke. Circulating markers of endothelial dysfunction and inflammation can identify patients at risk of stroke^43^ and endothelial dysfunction occurs in patients with acute stroke^44^. Structural alterations on MRI, indicative of small vessel disease, include white matter hyperintensities, lacunes, microbleeds and perivascular spaces and are associated with an increased risk of ischemic stroke^16^. There is a link between abnormalities in the eye and stroke, based on observations that altered retinal vessel function, diameter and geometry are related to cardiovascular disease^8,17^, stroke^18^ and recurrent stroke^19^.

Loss of cells with migration and increased size of neighboring cells and a loss of their hexagonal shape, leading to increased polymegathism and pleomorphism, respectively, characterize corneal endothelial cell pathology. However, these changes are inconsistent and vary in different conditions. We show a reduction in corneal endothelial cell density and an increase in size, but no change in polymegathism or pleomorphism. A recent study in patients with Type 2 diabetes has shown a reduction in endothelial cell density and increased polymegathism, but no change in pleomorphism^24^. In a study of children with Type 1 diabetes, polymegathism was increased, but pleomorphism was reduced^25^. In subjects with HIV, endothelial cell density was preserved, but polymegathism was increased^45^. In the present study we also show no difference in endothelial cell morphology between patients with and without diabetes, but an association with triglycerides diastolic blood pressure and HDL. Of relevance, metabolic syndrome, characterized by raised triglycerides and blood pressure and a low HDL, is an important risk factor for stroke^46^. Triglycerides were also the only lipid component to confer an increased risk of stroke in the prospective EPIC-Heidelberg cohort^47^.

This study has several limitations including the modest number of patients with mild ischemic stroke and we did not include other types of stroke. Nevertheless, we show corneal nerve loss and an alteration in corneal endothelial cell morphology in patients with acute ischemic stroke. Larger, longitudinal studies assessing corneal endothelial cell and nerve fibre morphology in those at risk of stroke and in relation to therapies to reduce risk factors for stroke are warranted to establish the clinical utility of corneal confocal microscopy in ischemic stroke.

## Methods

### Subjects

This study was a prospective, non-randomized clinical study. 146 patients underwent CCM within the first week (most within three days) of admission for an acute ischemic stroke. Stroke was confirmed clinically and radiologically by a neurologist subspecialized in stroke, based on WHO criteria^48^. Patients underwent assessment of the NIHSS (National Institutes of Health Stroke Scale) on admission. It allows grading of the severity of stroke into minor stroke (1–4 score), moderate stroke (5–15 score), moderate to severe stroke (16–20 score) and severe stroke (21–42 score). We could not undertake CCM in participants with major weakness; therefore only patients with mild stroke were examined.

Exclusion criteria included patients with intracerebral hemorrhage, a known history of eye trauma or surgery, any corneal or anterior segment pathology including neurotrophic keratitis, trigeminal neuralgia, keratoconus, high refractive error, dry eye, contact lens wear, Fuchs corneal dystrophy, posterior corneal dystrophy and glaucoma. Age-matched healthy control participants (n = 18) were recruited and assessed from Rumailah Hospital and Hamad General Hospital in Doha, Qatar.

This study adhered to the tenets of the declaration of Helsinki and was approved by the Institutional Review Board of Weill Cornell Medicine (15–00021) and Hamad General Hospital (15304/15). Informed, written consent was obtained from all patients/guardians before participation in the study. Clinical demographic parameters, blood pressure, HbA_1c_, total cholesterol, HDL, LDL and triglycerides were assessed on admission.

### Corneal Confocal Microscopy

All patients underwent CCM (Heidelberg Retinal Tomograph III Rostock Cornea Module, Heidelberg Engineering GmbH, Heidelberg, Germany). This device uses a 670 nm wavelength helium neon diode laser, which is a class I laser and therefore does not pose any ocular safety hazard. A 63x objective lens with a numerical aperture of 0.9 and a working distance, relative to the applanating cap (TomoCap^©^, Heidelberg Engineering GmbH, Heidelberg, Germany) of 0.0 to 3.0 mm is used. The size of each two-dimensional image produced is 384 μm × 384 μm with a 15° × 15° field of view and 10 μm/pixel transverse optical resolution. To perform the CCM examination, local anesthetic (0.4% benoxinate hydrochloride, Chauvin Pharmaceuticals, Chefaro, UK) was used to anaesthetize each eye and Viscotears (Carbomer 980, 0.2%, Novartis, UK) were used as the coupling agent between the cornea and the applanating cap. All patients were asked to fixate on an outer fixation light throughout the CCM scan and a CCD camera was used to correctly position the applanating cap onto the cornea. The examination took approximately 10 minutes for both eyes and was undertaken by experienced examiners (AK, GP, HA and INP), masked from the subject’s clinical status. Images of the endothelial cells and subbasal corneal nerves were captured using the “section” mode.

### Image Analysis

Corneal endothelial cell morphology was undertaken in 2-3 representative central images from each eye based on the depth (endothelial cell layer), focus (sharp focused images) and position (central cornea), with a frame size of at least 25%^49^. The image analysis was performed blindly without the investigator being aware of whether the images were from a control subject or patient with stroke. Each image was exported to a real-time automated image analysis system (Corneal Endothelium Analysis System (CEAS))^50^. A central region of interest (ROI) was traced for each image to identify the optimal area for quantification, avoiding peripheral darker areas. The CEAS system consists of a cell segmentation and morphometric parameter quantification stage. The former stage can be further divided into two steps: a pre-processing step and cell contour detection step. In the pre-processing step an FFT-Band-pass filter is applied to reduce noise and enhance image quality, followed by the detection of all endothelial cells in the image using a watershed transform and a Voronoi tessellation approach. A number of clinically useful features were extracted from the segmented endothelial cell images in an automated and objective manner to accurately describe the health of the corneal endothelium and include: Mean Endothelial Cell Density (ECD) (cell/mm^2^), Mean Endothelial Cell Area (ECA) (µm^2^), Mean Endothelial Cell Perimeter (ECP) (µm), polymegathism (%) and pleomorphism (%)^51^ (Fig. 1). Polymegathism (coefficient of variation) was defined as the standard deviation of the cell area divided by the mean cell area. Pleomorphism was defined as the hexagonality coefficient. The mean SD of the number of cells analysed per image was 136.38+/−61.22.

6 images/subject were selected for corneal nerve image analysis^52^. All CCM images were analyzed using validated, purpose-written software (CCMetrics^®^, M. A. Dabbah, ISBE, University of Manchester, Manchester, UK)^52^. Corneal nerve fiber density (CNFD) (no./mm^2^), corneal nerve fiber branch density (CNBD) (no./mm^2^) and corneal nerve fiber length (CNFL) (mm/mm^2^) were manually quantified.

### Statistical analysis

All statistical analysis was carried out using IBM SPSS Statistics software Version 24. Normality of the distribution of data was examined using the Kolmogorov-Smirnov test, and by visual inspection of the histogram and a normal Q-Q plot. Data is expressed as the mean ± standard error (Table 1). Statistical justification for the number of participants was based on a power analysis using the freeware program G*Power version 3.0.10 for α (type 1 error) of 0.05 and power (1 − type 2 error) of 0.80 using corneal nerve fibre density mean (37.12 vs 29.18) and standard deviation (8.35 and 7.16) comparing healthy controls to patients with stroke^32^.

The statistical distribution of healthy controls and patients with acute ischemic stroke and between stroke patients with and without diabetes was compared using the unpaired t test (2-tailed) (normally distributed variables) and Mann-Whitney test (non-normally distributed variables). Bonferroni correction was applied to control for multiple testing where *P* = 0.006, based on eight independent observations.

To investigate the association between risk factors for stroke and corneal endothelial cell parameters, Pearson correlation was performed and multiple linear regression was conducted to assess the association between endothelial cell abnormalities and co-variates. Significance level was set at *P* = 0.05. Prism 6 (version 6.0 g, Graphpad software Inc., CA, USA) was used to plot the graphs.

## Electronic supplementary material

Supplementary Table S1 and S2

## Data Availability

The datasets generated during and/or analysed during the current study are available from the corresponding author on reasonable request.
