# Supplementary material for: Corneal Confocal Microscopy detects a Reduction in Corneal Endothelial Cells and Nerve Fibres in Patients with Acute Ischemic Stroke
Source: Sci Rep. 2018 Nov 26;8:17333. doi: 10.1038/s41598-018-35298-3 (PMC6255787; doi:10.1038/s41598-018-35298-3)
Supplement: Supplementary file 1 — Supplementary Table S1 and S2 [file 41598_2018_35298_MOESM1_ESM.docx]

**Corneal Confocal Microscopy detects a Reduction in Corneal Endothelial Cells and Nerve Fibres in Patients with Acute Ischemic Stroke**

Adnan Khan^1^, PhD; Saadat Kamran^2^, MD; Naveed Akhtar^2^, MD; Georgios Ponirakis^1^, MPhil; Hamad Al-Muhannadi^1^, Ioannis N. Petropoulos^1^, PhD; Shumoos Al-Fahdawi^3^, PhD, Rami Qahwaji^3^, PhD, Faheem Sartaj^2^, MBBS; Blessy Babu^2^, MBBS, Muhammad Faisal Wadiwala^2^, MD, Ashfaq Shuaib^2, 4^, MD; Rayaz A. Malik^1^ MD, PhD

Supplementary Table 1. Correlation between endothelial cell and corneal nerve parameters in patients with ischemic stroke without diabetes, with significant values in bold. ECD (Endothelial Cell Density), ECA (Endothelial Cell Area), ECP (Endothelial Cell Perimeter), CNFD (Corneal nerve fibre density), CNBD (Corneal nerve branch density), CNFL (Corneal nerve fibre length).

| Variables | CNFD | CNFL | CNBD |
| --- | --- | --- | --- |
| Endothelial Cell Density  Coefficient (r)  *P* | **0.208**  **(0.042)** | **0.188**  **(0.067)** | 0.130  (0.208) |
| Endothelial Cell Area  Coefficient (r)  *P* | **–0.241**  **(0.018)** | **–0.207**  **(0.037)** | –0.152  (0.139) |
| Endothelial Cell Perimeter  Coefficient (r)  *P* | **–0.236**  **(0.021)** | **–0.216**  **(0.035)** | –0.155  (0.131) |
| Polymegathism  Coefficient (r)  *P* | 0.062  (0.548) | 0.115  (0.263) | 0.082  (0.430) |
| Pleomorphism  Coefficient (r)  *P* | –0.011  (0.916) | –0.009  (0.928) | –0.001  (0.995) |

Supplementary Table 2. Correlation between endothelial cell and corneal nerve parameters in patients with ischemic stroke and diabetes, with significant values in bold. ECD (Endothelial Cell Density), ECA (Endothelial Cell Area), ECP (Endothelial Cell Perimeter), CNFD (Corneal nerve fibre density), CNBD (Corneal nerve branch density), CNFL (Corneal nerve fibre length).

| Variables | CNFD | CNFL | CNBD |
| --- | --- | --- | --- |
| Endothelial Cell Density  Coefficient (r)  *P* | 0.090  (0.540) | 0.204  (0.159) | 0.080  (0.583) |
| Endothelial Cell Area  Coefficient (r)  *P* | –0.110  (0.452) | –0.182  (0.210) | –0.073  (0.620) |
| Endothelial Cell Perimeter  Coefficient (r)  *P* | –0.133  (0.363) | –0.212  (0.144) | –0.103  (0.483) |
| Polymegathism  Coefficient (r)  *P* | **–0.373**  **(0.008)** | **–0.296**  **(0.039)** | **–0.334**  **(0.019)** |
| Pleomorphism  Coefficient (r)  *P* | **0.309**  **(0.031)** | 0.193  (0.183) | 0.259  (0.073) |
